# Supplementary material for: In Vitro High-Throughput Toxicological Assessment of Nanoplastics
Source: Nanomaterials (Basel). 2022 Jun 7;12(12):1947. doi: 10.3390/nano12121947 (PMC9230863; doi:10.3390/nano12121947)
Supplement: Supplementary file 1 [file nanomaterials-12-01947-s001.zip › nanomaterials-1723499-supplementary.pdf]

nanomaterials

Article

In Vitro High-Throughput Toxicological Assessment of Nanoplastics

Valentina Tolardo [1,2], Davide Magrì [3], Francesco Fumagalli [3], Domenico Cassano [3], Athanassia Athanassiou [1], Despina Fragouli [1] and Sabrina Gioria [3,*]

1 Smart Materials, Istituto Italiano di Tecnologia, Via Morego, 30, 16163 Genova, Italy; valentina.tolardo@iit.it (V.T.); athanassia.athanassiou@iit.it (A.A.); despina.fragouli@iit.it (D.F.)
2 Department of Informatics, Bioengineering, Robotics and Systems Engineering, University of Genova, Via All' Opera Pia, 13, 16145 Genova, Italy
3 European Commission, Joint Research Centre (JRC), 21027 Ispra, Italy; davide.magri.mail@gmail.com (D.M.); francesco-sirio.fumagalli@ec.europa.eu (F.F.); domenico.cassano@sns.it (D.C.)
* Correspondence: sabrina.gioria@ec.europa.eu

Supplementary

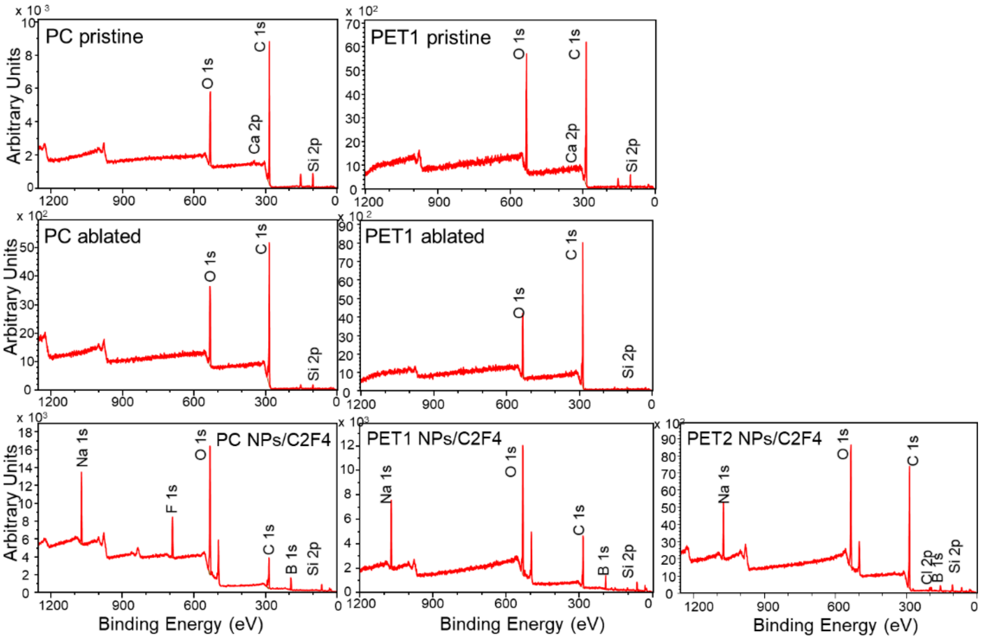

Figure S1. Survey XPS spectra of the pristine polymer and of the as-synthesized NPs with the respective elemental composition.

Table S1. Percentage of the respective elemental composition of survey spectra.

	**Binding Energy**	**PC pristine**	**PC ablated**	**PC NPs/C2F4**
**C 1s**	284.7 eV	78.60%	81.44%	29.66%
**O 1s**	532.2 eV	16.17%	16.65%	45.37%
**Si 2p**	102.2 eV	5.22%	1.91%	0.40%
**B 1s**	192.7 eV			14.52%
**Ca 2p**	347.3 eV	0.31%		
**F 1s**	689.7 eV			8.9%
**Na 1s**	1071.16 eV			10.05%
	**Binding Energy**	**PET1 pristine**	**PET1 ablated**	**PET1 NPs/C2F4**
**C 1s**	284.7 eV	74.64%	81.55%	57.59%
**O 1s**	532.2 eV	23.39%	16.29%	33.72%
**Si 2p**	102.2 eV	1.86%	2.17%	2.87%
**B 1s**	192.7 eV			10.39%
**Ca 2p**		0.12%		
**Na 1s**	1071.16 eV			3.49%
	**Binding Energy**			**PET2 NPs/C2F4**
**C 1s**	284.7 eV			66.71%
**O 1s**	532.2 eV			20.70%
**Si 2p**	102.2 eV			3.29%
**B 1s**	192.7 eV			2.94%
**Cl 2p**	197.8 eV			0.75%
**Na 1s**	1071.16 eV			5.61%

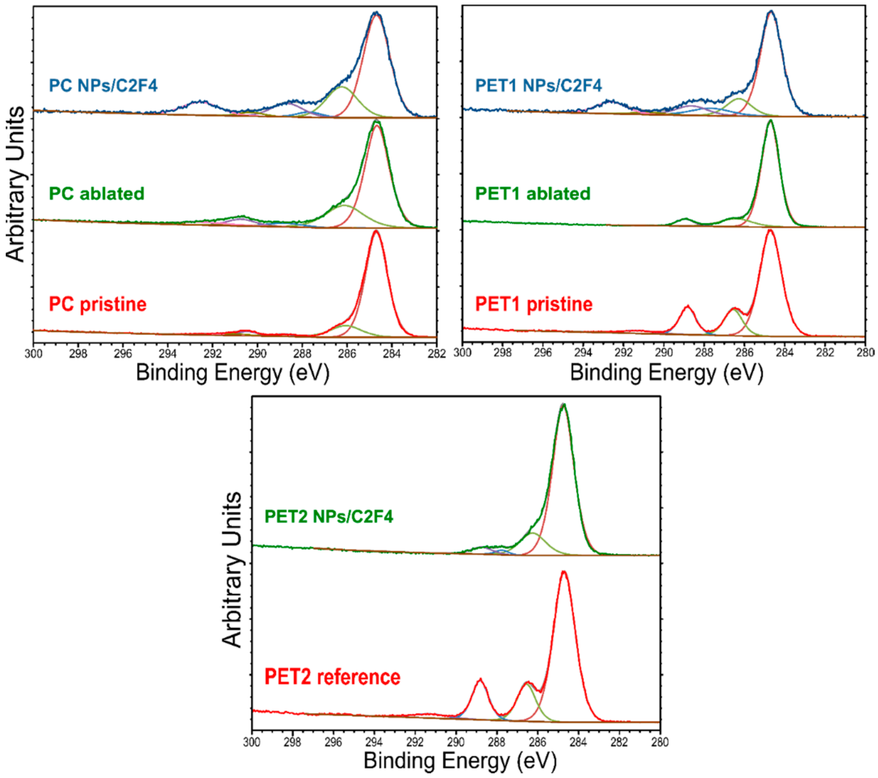

Figure S2. XPS C1s high resolution spectra of the pristine polymer and of the as-synthesized NPs with the corresponding deconvolutions.

Table S2. Percentage of corresponding deconvolutions results of C1s spectra.

<table>
<tr><td>C 1s</td><td>Binding Energy</td><td>PC pristine</td><td>PC ablated</td><td>PC NPs/C2F4</td></tr>
<tr><td>C-C (ar)</td><td>284.6 eV</td><td>60.93%</td><td>48.07%</td><td rowspan="2">58.38%</td></tr>
<tr><td>C-C (al)</td><td>285.1 eV</td><td>22.53%</td><td>17.77%</td></tr>
<tr><td>C-O-C, C-OH</td><td>286.3 eV</td><td>8.67%</td><td>19.22%</td><td>17.79%</td></tr>
<tr><td>C=O</td><td>287.7 eV</td><td></td><td></td><td>3.27%</td></tr>
<tr><td>O=C-O</td><td>288.7 eV</td><td>1.87%</td><td>4.01%</td><td>8.02%</td></tr>
<tr><td>O=C(-O)2</td><td>290.4 eV</td><td>2.61%</td><td>3.73%</td><td>2.02%</td></tr>
<tr><td>C-C sh.-up (I)</td><td>291.3 eV</td><td>2.17%</td><td>2.27%</td><td></td></tr>
<tr><td>C-C sh.-up (II)</td><td>292.5 eV</td><td>1.23%</td><td>4.94%</td><td></td></tr>
<tr><td>C-F2</td><td>292.5 eV</td><td></td><td></td><td>10.52%</td></tr>
<tr><td>C 1s</td><td>Binding Energy</td><td>PET1 pristine</td><td>PET1 ablated</td><td>PET1 NPs/C2F4</td></tr>
<tr><td>C-C (ar)</td><td>284.6 eV</td><td>37.31%</td><td rowspan="2">78.08%</td><td rowspan="2">58.92%</td></tr>
<tr><td>C-C (al)</td><td>285.1 eV</td><td>24.62%</td></tr>
<tr><td>C-O-C, C-OH</td><td>286.3 eV</td><td>15.68%</td><td>13.50%</td><td>16.82%</td></tr>
<tr><td>C=O</td><td>287.7 eV</td><td></td><td></td><td>3.96%</td></tr>
<tr><td>O=C-O</td><td>288.7 eV</td><td>15.42%</td><td>6.31%</td><td>10.92%</td></tr>
<tr><td>C-C sh.-up (I)</td><td>291.3 eV</td><td>5.54%</td><td>1.52%</td><td>2.84%</td></tr>
</table>

C-C sh.-up (II)	292.5 eV	1.43%	0.60%	
**C 1s**	**Binding Energy**	**PET2 reference**		**PET2 NPs/C2F4**
C-C (ar)	284.6 eV	37.31%		82.94%
C-C (al)	285.1 eV	24.62%		
C-O-C, C-OH	286.3 eV	15.68%		12.81%
C=O	287.7 eV			
O=C-O	288.7 eV	15.42%		4.25%
C-C sh.-up (I)	291.3 eV	5.54%		
C-C sh.-up (II)	292.5 eV	1.43%		

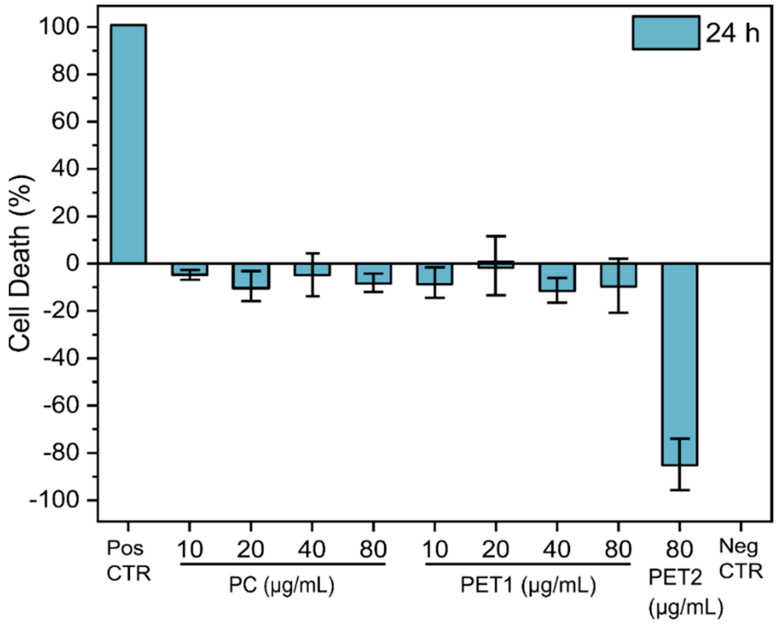

Figure S3. HepG2 cell viability after exposure to NPs at the concentration of 10, 20, 40, 80 μg/mL for 24 or 48 h. In the case of PET2, data are represented only at 80 μg/mL (LDH assay). Data are reported as % of cell death (average of three independent experiments ± SD).

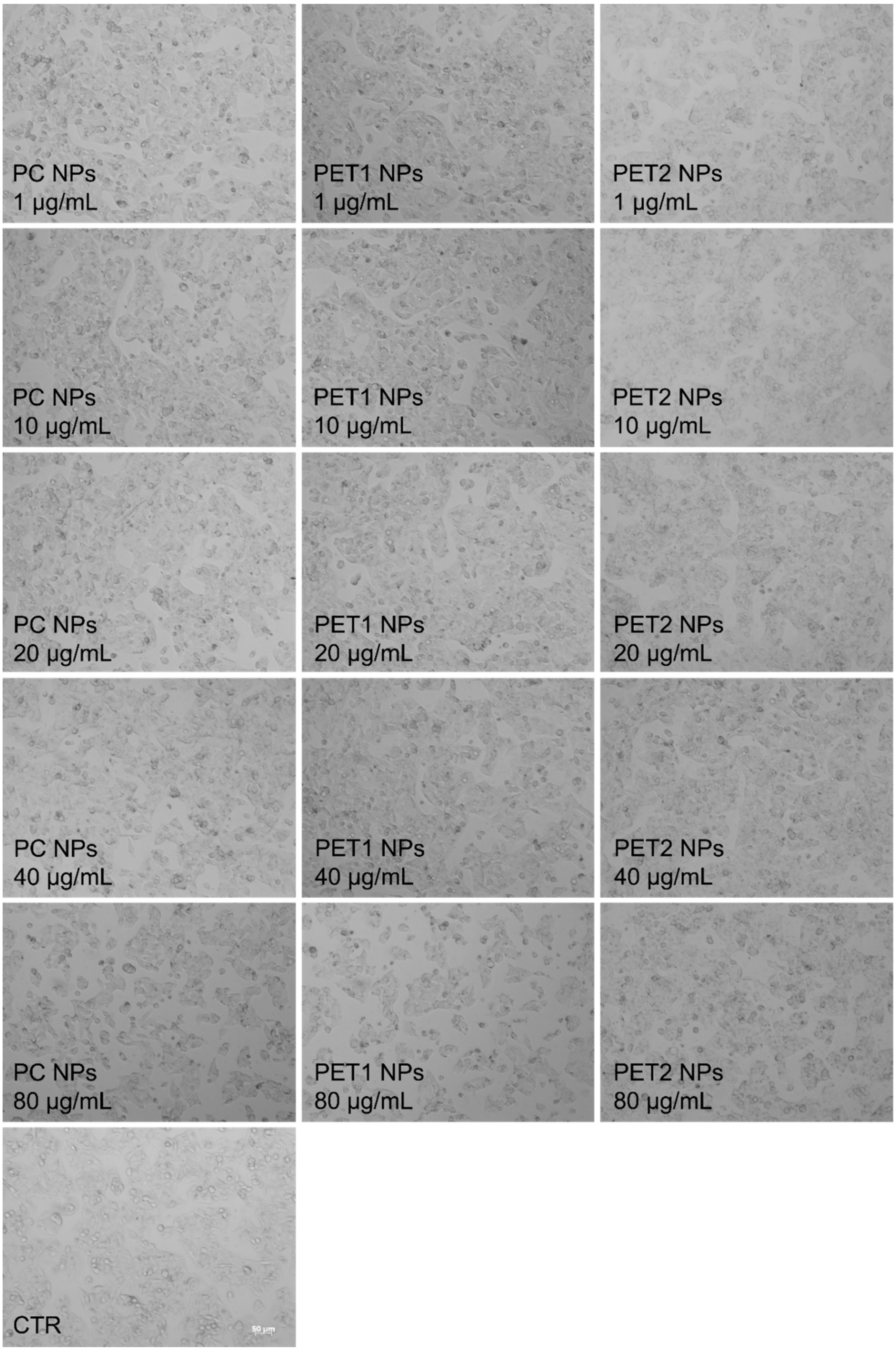

Figure S4. Representative morphological images of HePG2 cells after different treatments for 24 h. Scale bar at 50 μm for all images.

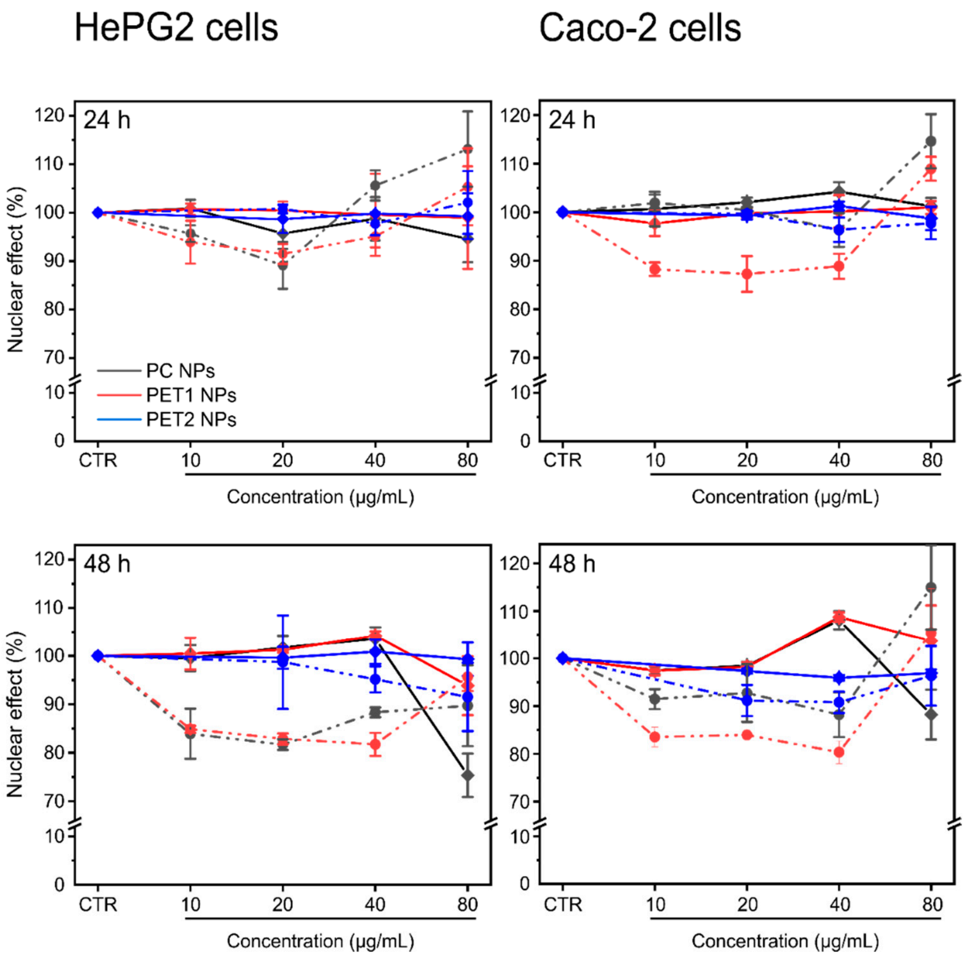

Figure S5. NPs effects on HePG2 or Caco-2 cells on nuclear size (line) and nuclear intensity (dash dot). Cells were exposed to PC, PET1 or PET2 NPs at concentrations between 10 and 80 µg/mL for 24 or 48 h, stained with Hoechst 33342 and analyzed by HCI. Data are expressed as % nuclear effect normalized to the control (untreated cells) and reported as mean of three independent experiments ± SD.
